# Acetylcholine activates a regenerative vasodilator mechanism that is sensitive to nitric oxide production

**DOI:** 10.3389/fphys.2025.1569167

**Published:** 2025-05-30

**Authors:** Nelson P. Barrera, Mónica Márquez, Matías Muñoz-Uribe, Xavier F. Figueroa

**Affiliations:** Facultad de Ciencias Biológicas, Pontificia Universidad Católica de Chile, Santiago, Chile

**Keywords:** conducted vasodilation, endothelial cells, nitric oxide, resting arteriolar diameter, mouse cremaster arterioles, endothelial nitric oxide synthase, endothelium-derived hyperpolarization, acetylcholine

## Abstract

**Introduction:**

The conduction of changes in the diameter of arterioles plays an important role in the coordination of the blood flow distribution. The endothelium regulates vasomotor tone by generation of vasodilator signals, such as nitric oxide (NO) and endothelium-derived hyperpolarization (EDH). Endothelium-mediated vasodilator responses initiated in an arteriolar segment are conducted along the vessel length, which depends on the electrotonic spread of EDH signaling activated at the stimulation site, but, in contrast, the contribution of NO is controversial.

**Methods:**

We used the mouse cremaster muscle microcirculation *in vivo* to analyze the participation of NO in the mechanisms involved in the conducted vasodilation observed in response to the stimulation of a short arteriolar segment with a pulse of acetylcholine (ACh), an endothelium-dependent vasodilator, or S-nitroso-N-acetylpenicillamine (SNAP), an NO donor.

**Results:**

The response to ACh spread along the entire vessel showing only a slight decay and, in contrast, the dilation evoked by SNAP was restricted to the stimulation site, independently of the magnitude of the response. Blockade of NO production with 100 μM N^G^-nitro-L-arginine methyl ester (LNAME) or 100 µM N^G^-nitro-L-arginine (L-NA) reduced the arteriolar resting diameter by 10%–12%, but the combined application of both blockers enhanced the basal vasoconstrictor tone by ∼38% and inhibited the local (∼45%) and conducted (∼20%–35%) responses initiated by ACh. Interestingly, the conduction of ACh-induced vasodilation increased along the vessel length in the presence of L-NAME and L-NA. In addition, blockade of endothelial cell hyperpolarization exclusively at the stimulation site through microsuperfusion of tetraethylammonium (TEA) inhibited the local vasodilation, but not the conduction of the response.

**Conclusion:**

These results suggest that ACh activates an NO sensitive mechanism of regenerative propagation of vasodilator responses, which contributes to our understanding of microvascular function and the complex integration of endothelial signaling pathways in the coordination of the blood flow distribution.

## Introduction

Much of the resistance to blood flow is located in feed arteries and arterioles (i.e., resistance arteries) in the microcirculation. Changes in the diameter of these vessels play a central role in the control of systemic arterial blood pressure and blood flow distribution (Mulvany and Aalkjaer, 1990; Segal et al., 2000; Segal, 2005). Blood vessels are complex, multicellular structures that must work as a unit to rapidly adjust the distribution of blood flow according to the changing metabolic demand of the cells of the surrounding tissue (Segal, 2005; Figueroa and Duling, 2009); consequently, an increase in the metabolic demand must be associated with a vasodilation-mediated increment in blood flow supply to the tissue. In addition to the response directly activated at the site of stimulation, changes in the diameter are also conducted along the length of resistance arteries, and conduction of vasomotor signals has emerged as an important physiological mechanism for coordinating vascular resistance within the microvascular bed, connecting the functions of the distal and proximal segments of the vasculature (Gustafsson and Holstein-Rathlou, 1999; Segal, 2005; Figueroa and Duling, 2008; Figueroa and Duling, 2009). Cells of the vessel wall are functionally connected via gap junctions, and conducted vasomotor responses are associated with the propagation of an electrical signal (Xia and Duling, 1995; Emerson and Segal, 2000). Conduction of the changes in the vessel diameter is the result of the electrotonic spread via gap junctions of the variations in membrane potential observed at the stimulation site. In this context, depolarization is associated with the conduction of vasoconstriction and hyperpolarization with the spread of vasodilation (Welsh and Segal, 1998; Emerson and Segal, 2000; Figueroa et al., 2004). It is noteworthy that conduction of vasodilator responses is not only important for efficient coordination of the blood flow distribution, but it is also relevant in the control of arterial blood pressure since a decrease in the propagation of these responses results in exercise-induced hypertension (Morton et al., 2015).

Although the magnitude of the vessel diameter is determined by the degree of smooth muscle contraction (i.e., vasomotor tone), Ca^2+^-dependent production of vasodilator signals by endothelial cells plays a critical role in the control of vascular resistance to blood flow along the time (Dora et al., 1997; Figueroa and Duling, 2009; Lillo et al., 2018). Nitric oxide (NO) has widely been recognized as the primary endothelium-dependent vasodilator signal in large conduit vessels (Moncada and Higgs, 2018). However, in small-resistance arteries and arterioles, the relevance of a complementary vasodilator component associated with endothelial cell-mediated smooth muscle hyperpolarization was also identified (Félétou and Vanhoutte, 2009). This additional vasodilator signal was first thought to be a factor released by endothelial cells, but it is currently recognized that Ca^2+^-activated K^+^ (K_Ca_) channels of small (SK_Ca_) and intermediate (IK_Ca_) conductance play an important role in triggering this vasodilator signal by generating a hyperpolarization, which is transmitted from endothelial cells to smooth muscle cells through the gap junctions connecting these two cell types (i.e., myoendothelial gap junctions), leading to the classification of this vasodilator signaling as endothelium-derived hyperpolarization (EDH) (Félétou and Vanhoutte, 2013). It must be noted that, in blood vessels, SK_Ca_ and IK_Ca_ channels are only expressed in endothelial cells (Doughty et al., 1999; Crane et al., 2003; Grgic et al., 2005) and that vasodilator responses have been found to be conducted mainly through the endothelium (Figueroa et al., 2007; Figueroa and Duling, 2008).

NO is generated by the enzyme NO synthase (NOS), and of the three isoforms of NOS, endothelial isoform (eNOS) is expressed in the endothelium (Moncada and Higgs, 2018). As the vasodilation activated by NO production is mainly mediated by a reduction in the Ca^2+^ sensitivity of smooth muscle contractile machinery (Bolz et al., 1999; Bolz et al., 2003), the conduction of endothelium-dependent vasodilator responses triggered in a short arteriolar segment is thought to rely exclusively on the longitudinal spread along the vessel axis of the hyperpolarization elicited by EDH signaling, whereas NO only contributes to the local vasodilation observed at the stimulation site (Hoepfl et al., 2002; Welsh and Segal, 2017). However, NO may also be involved in the functional coordination of the vasomotor tone among the arterioles in the microcirculation since the conducted vasodilation observed in response to acetylcholine (ACh) is inhibited by histamine in an NO-dependent manner (Payne et al., 2004). Furthermore, after blocking the activity of the NO-synthesizing enzyme, conduction was not only restored in control animals but also enhanced in PECAM-1-knockout mice (i.e., CD31), suggesting that in addition to contributing to the local response, NO may also be involved in the regulation of the mechanism of conduction of the vasodilator signal (Payne et al., 2004), but this proposal has not been directly evaluated.

Based on the above-described findings, we hypothesized that NO participates in the regulation of the mechanism involved in the coordination of changes in the diameter of resistance arteries in the microcirculation by controlling the conduction of vasodilator responses. We evaluated the relevance of NO production in the tonic vasodilator component observed in resting diameter and in the magnitude of conducted vasodilator signals activated by ACh. Our findings indicate that stimulation with ACh triggers the initiation of a regenerative propagation mechanism of a vasodilator signal coupled to the activation of NO production and EDH signaling along the arteriolar length. In addition, the ACh-evoked regenerative vasodilator mechanism is sensitive to NO, which, consequently, works as negative feedback signaling on the conducted vasodilation.

## Materials and methods

Male C57 Bl/6 (wild type) mice weighing between 22 and 28 g were used. Mice were bred and maintained in the Research Animal Facility of the Pontificia Universidad Católica de Chile, and all studies were approved by the Institutional Bioethics Committee (protocol ID 210422002). Experiments were conducted according to the guidelines of the Helsinki Declaration and the National Institutes of Health Guide for the Care and Use of Laboratory Animals (NIH Publications No. 8523, revised 2011). All efforts were made to minimize the suffering and the number of animals used.

### Mouse cremaster preparation

Mice were anesthetized with pentobarbital sodium (40 mg/kg, i.p., diluted in isotonic saline to 5 mg/mL), placed on a Plexiglas board, and the cremaster muscle microcirculation was prepared as described previously (Figueroa and Duling, 2008). The right cremaster muscle was exposed and opened by a longitudinal incision on its ventral surface, and the testis and epididymis were excised after ligating the supply vessels. The cremaster was pinned out on a silicone rubber pedestal, and the mouse was placed on a Gibraltar Platform coupled to an Olympus microscope (BX 50WI). The body temperature was maintained at 35°C–36°C using a heating pad, and the cremaster muscle was continuously superfused at 3 mL/min with a bicarbonate-buffered saline solution (mM: 131.9 NaCl, 4.7 KCl, 2.0 CaCl_2_, 1.2 MgSO_4_, and 20.0 NaHCO_3_) kept at 35°C and equilibrated with 95% N_2_–5% CO_2_ to yield a pH of 7.35–7.45. The preparation was allowed to stabilize for 45–60 min before starting the experiment. Supplemental doses of dilute anesthetic in isotonic saline (10 mg/kg, i.p.) were administrated as appropriate throughout the experiment. At the end of the experiment, the animals were euthanized by application of an anesthetic overdose.

### Vessel diameters

The cremaster muscle was transilluminated, and the microscopic image was acquired using a video camera (Dage-MTI Series 65, IN) and then displayed on a monitor. The inner diameters of the arterioles were continuously measured using Diamtrak software (Figueroa et al., 2007; Figueroa and Duling, 2008).

To evaluate the conduction of vasomotor responses along the vessel length, the stimulation was restricted to a small segment (∼70 µm) of the arterioles through the application of 10 μM ACh, 10 µM S-nitroso-N-acetylpenicillamine (SNAP), or 1 µM calcitonin gene-related peptide (alpha isoform, α-CGRP) via a pressure-pulse ejection (10–15 psi) from a micropipette (inner diameter of 3–4 µm). The durations of the pressure-pulse ejections of ACh and α-CGRP were set to induce a local vasodilation of ∼50% (ACh: 400 ms and α-CGRP: 500 ms) and also 30% in one group of arterioles stimulated with ACh (200 ms). In the case of the SNAP, the stimulation period was adjusted to evoke a response of a similar magnitude to that of the NO-mediated vasodilator component activated by ACh (a pressure-pulse duration of 300 ms) and, in one group of experiments, the length of the pulse of the SNAP was extended to 700 ms to evaluate the conduction of a larger vasodilation, as that attained in response to ACh.

### Experimental protocols

In all experiments, conduction of the responses was analyzed in the arterioles of the second and third orders throughout a vessel segment that did not show evident branches, which prevents the potential dissipation of the vasodilator signal, and changes in diameter were measured first at the stimulation site (local) and then at locations 500, 1,000, and 2,000 µm upstream in four separate stimuli. The maximal diameter was estimated during superfusion of 1 mM adenosine after completion of the experimental protocol, and variations in the diameter were expressed as a percentage of the maximal dilation possible (% Maximum), using the following equation: (*D*_st_−*D*_cont_)/(*D*_max_−*D*_cont_) × 100, where *D*_st_ is the diameter after stimulation, *D*_cont_ is the diameter before stimulation (control diameter), and *D*_max_ is the maximal diameter. In addition, the mechanical length constant (λ) was also calculated in some groups of experiments, as described previously (Xia and Duling, 1995): D_1_/D_2_ = e^−x/λ^, where D represents the diameter change at two different points in response to the stimulation and x is the distance between D_1_ and D_2_. The length constant corresponds to the distance at which the magnitude of the response observed at the stimulation site (local) decreased to 37%.

#### Focal application of tetraethylammonium (TEA)

To block the activation of EDH signaling exclusively in the vessel segment to be stimulated with ACh, the tip (inner diameter ∼10 µm) of a micropipette filled with MOPS-buffered (pH 7.4) saline solution containing 100 mM tetraethylammonium (TEA) was positioned above the stimulation site of the arteriole (local), and the blocker was ejected by pressure during 10–15 min prior to ACh application.

#### Global blockade of NO production

Two NOS blockers were used, N^G^-nitro-L-arginine methyl ester (L-NAME) and N^G^-nitro-L-arginine (L-NA), which were applied through the superfusion solution to achieve a homogenous distribution of the inhibitors in the preparation and, thereby, prevent the production of NO along the whole arterioles. L-NAME and L-NA are the most frequently used NOS inhibitors in vascular biology, and because the inhibitory effect of these blockers is time-dependent, the responses were assessed after 45 min of their topical application (Figueroa et al., 2001; 2002). In addition, as the IC_50_ of these blockers is lower than 1 µM (Boer et al., 2000; Alderton et al., 2001) and a maximal effect can be observed with 30 µM (Boer et al., 2000; Figueroa et al., 2001; Figueroa et al., 2002), a supramaximal concentration (100 µM) of L-NAME or L-NA was used to achieve the largest possible inhibitory effect. However, although analogs of L-arginine, such as L-NAME and L-NA, have been demonstrated to be good pharmacological tools to evaluate the relevance of NO production in the regulation of vascular function, both blockers only achieve a partial inhibition of endothelial cell-mediated NO production (Cohen et al., 1997; Støen et al., 2003). Furthermore, L-NAME is 30–100-fold less potent than L-NA (Cohen et al., 1997; Boer et al., 2000), suggesting that L-NAME and L-NA may target distinct eNOS pools that contribute a different concentration of NO. Then, the effects of the combined application of both blockers (100 µM each) were also evaluated.

### Chemicals

All chemicals of analytical grade were obtained from Merck (Darmstadt, Germany). In addition, adenosine, ACh, L-NAME, L-NA, TEA, and MOPS were purchased from Sigma Chemical Co., (St. Louis, MO, United States). SNAP was obtained from Calbiochem (La Jolla, CA, United States), and α-CGRP was purchased from Bachem (Torrance, CA, United States). SNAP was dissolved in DMSO and then diluted in the buffer solution to the final working concentration. Control experiments confirmed that the application of the vehicle of SNAP (DMSO) did not have effect *per se* (data not shown).

### Statistical analysis

Results are presented as mean ± S.E.M. The differences between the responses of two groups (control vs. treatment) were analyzed through paired Student’s *t*-test, and multiple comparisons between several experimental groups and the control group were made using one-way ANOVA plus Newman–Keuls *post hoc* test. *p* < 0.05 was considered significant.

## Results

Cremasteric arterioles of second and third branching orders were analyzed. The maximum diameter of these arterioles ranged from 25.2 to 56.6 µm, and the mean resting diameter was 20.5 ± 1.2 µm (*n* = 28), reflecting the prominent degree of the vasomotor tone developed by these arterioles (49.1% ± 2.5%), which remained stable over time in resting conditions. The level of vasomotor tone *in vivo* is tonically controlled by the endothelium through NO production (de Wit et al., 1997; de Wit et al., 1998; Figueroa et al., 2001), but the magnitude of the NO-dependent vasodilator component in resting conditions is contended.

### Contribution of tonic NO production to vasomotor tone

**FIGURE 1:**
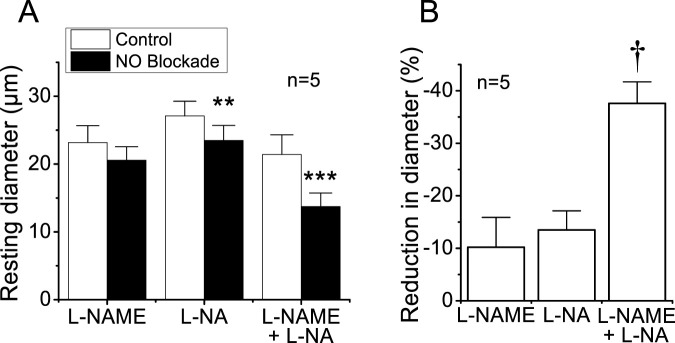
Reduction in the diameter of cremasteric arterioles observed in response to the blockade of NO production. Cremaster muscle microcirculation was treated for 45 min with 100 μM L-NAME, 100 μM L-NA, or the combination of both blockers (100 µM each) of the enzyme NOS, and the changes in diameter were evaluated 45 min thereafter **(A)**. In addition, the percentage of reduction in vessel diameter attained in the presence of the blockers is also shown **(B)**. Note that the combined application of L-NAME and L-NA evoked an effect almost three-fold larger than that observed with each blocker alone. **P < 0.01 and ***P < 0.001 vs. control by paired Student’s *t*-test. †*P* < 0.05 vs. L-NAME or L-NA by one-way ANOVA plus Newman–Keuls *post hoc* test.

To evaluate the importance of endothelium-mediated NO signaling in the tonic control of vasomotor tone, we measured the changes in the basal diameter observed after the treatment for 45 min with the NOS blockers L-NAME, L-NA, or the combination of both. Application of either 100 μM L-NAME or 100 μM L-NA elicited a small, consistent decrease in the diameter (Figure 1A), but only the effect of L-NA reached significance. Interestingly, in contrast to the modest effect of L-NAME or L-NA alone, the combined application of both blockers resulted in a 37.6% ± 4.1% of reduction in the diameter of the arterioles (Figure 1A), which was almost three times more prominent than that observed with each blocker separately (Figure 1B), indicating the presence of a synergistic effect between these two inhibitors and highlighting the relevance of NO production in the control of vascular function.

### Contribution of NO production to ACh-activated conducted vasodilation

**FIGURE 2:**
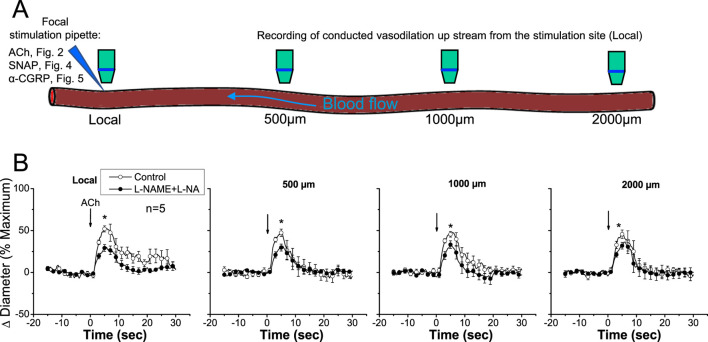
Time course of the local and conducted vasodilation induced by ACh under control conditions and after blocking NO production. **(A)** Schematic representation of experimental procedures. Local, 500 μm, 1,000 μm, and 2,000 µm denote sites at which vasomotor responses were assessed. References to other figures indicate that the same experimental procedure was applied in those cases. **(B)** ACh was ejected by a pressure pulse via a micropipette in order to stimulate a short segment of the cremasteric arterioles, and the vasodilator response was analyzed at the stimulation site (local) and at 500, 1,000, and 2,000 µm upstream. The vasodilator responses initiated by ACh were evaluated before and after blocking NO production through the application of the combination of L-NAME and L-NA. Note that the changes in the diameter do not decay along the vessel length in the presence of the combination of L-NAME and L-NA. Arrows indicate the time at which the stimulus was applied. *P < 0.05 vs. control by one-way ANOVA plus Newman–Keuls *post hoc* test.

**FIGURE 3:**
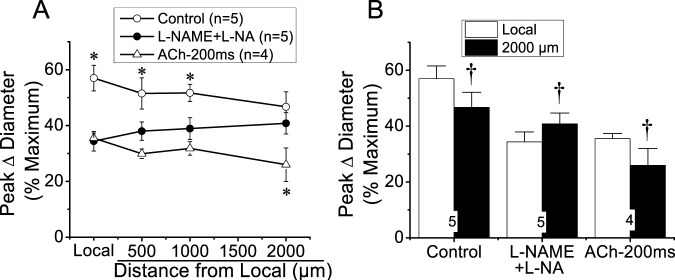
Analysis of the effect of NO production blockade on the conducted vasodilation activated by ACh. A short segment of the arteriole was stimulated with a pulse (400 ms) of 10 µM ACh ejected by pressure pulse from a micropipette. **(A)** The analysis of the maximal vasodilator response induced by ACh in the experiments described in Figure 2B is shown. The vasodilation was evaluated before (control) and after the blockade of NO production with the combination of 100 μM L-NAME and 100 μM L-NA. In addition, conduction of the response induced by a shorter pressure pulse of ACh (ACh-200 ms) is also assessed. **(B)** Change in the magnitude of the vasodilation evoked by ACh in the stimulation site (local) compared with that observed 2,000 µm upstream. Note that in the presence of L-NAME and L-NA, the vasodilation increased, instead of decaying, along the vessel length. *P < 0.05 vs. L-NAME + L-NA by one-way ANOVA plus Newman–Keuls *post hoc* test. †*P* < 0.05 vs. local by paired Student’s *t*-test.

Stimulation of a short arteriolar segment with a pulse of ACh induced a rapid and transient vasodilator response that reached a peak after ∼5 s and gradually returned to control diameter within 10–20 s (Figure 2). The response to ACh was not restricted to the stimulation site (i.e., local site), but it was rapidly propagated along the entire arteriole, showing only a slight decrease in the magnitude (Figure 2), mainly during the first 500 µm and then beyond the 1,000 µm conducted site (Figures 2, 3). Although the small decrease in the conducted vasodilation activated by ACh precludes the possibility of a precise calculation of the mechanical length constant (Xia and Duling, 1995) of the response, this parameter may be estimated if an exponential decay is assumed, which suggests that the mechanical length constant of the response to ACh (11.1 ± 2.5 mm) was much higher than the electrical length constant (0.9–1.6 mm) determined by current injection in arterioles *in vitro* or *in vivo* (Hirst and Neild, 1978; Hirst et al., 1997; Emerson et al., 2002). The small decrease in the amplitude of the ACh-activated vasodilator response along the length of the arteriole is not consistent with a simple electrotonic conduction of a hyperpolarizing signal (Figueroa and Duling, 2008) and rather suggests that a regenerative mechanism of propagation is involved in the process, as further supported by the blockade of NO production. Interestingly, the blockade of NO production with the combined application of L-NAME and L-NA not only reduced the magnitude of the ACh-induced vasodilation at the local site, as expected, but also inhibited the response achieved at the conducted sites (Figure 2). In addition, in contrast to the decay with the distance observed in control conditions (−22.9% ± 6.1% from local to 2,000 µm upstream), the conduction of the vasodilator response initiated by ACh was enhanced (18.4% ± 6.0% from local to 2,000 µm upstream; *P* < 0.008 vs. control conditions by paired Student’s *t*-test) in the presence of L-NAME and L-NA (Figures 2, 3A), and consequently, the vasodilation recorded at 2,000 µm was higher than that measured at the stimulation site (Figure 3B). Nevertheless, the effect of the inhibition of NO production on the response may be related to the decrease in the magnitude of ACh-induced vasodilation. Then, to evaluate this possibility, we reduced the pressure-pulse ejection of ACh to elicit a response of similar magnitude to that observed after blocking NO production. Under these conditions, the propagation of the vasodilation decreased along the length of the arterioles, showing exactly the same characteristics of that observed with the higher ACh stimulation under control conditions (Figure 3A), and the magnitude of the decay in the vasodilation from the ACh application site to the 2,000-µm conducted site was similar (Figure 3B), which confirms the participation of NO in the coordination of the changes in the vessel diameter.

**FIGURE 4:**
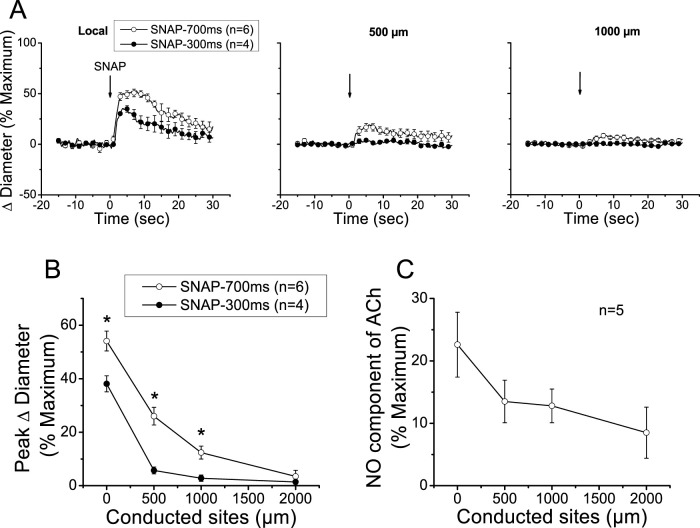
Analysis of the conducted vasodilation activated by direct stimulation with a pulse of NO. **(A)** Time course of the local and conducted vasodilation induced by 700 ms or 300 ms pressure pulse of 10 µM SNAP, an NO donor. Arrows indicate the time at which the stimulus was applied. **(B)** Maximal vasodilator response induced by SNAP observed at the stimulation site (local in panel **A**) and at locations 500, 1,000, and 2,000 µm upstream. **(C)** Analysis of the conduction of the NO-dependent vasodilator component of the response activated by ACh under control conditions shown in Figure 2. Note that, in contrast to the stimulation with SNAP, the ACh-activated NO-dependent vasodilator component exhibits only a moderate decay along the length of the arterioles, and the response can be observed up to the conducted site located at 2,000 µm from the stimulation site. *P < 0.05 vs. SNAP-700 ms by one-way ANOVA plus Newman–Keuls *post hoc* test.

The reduction in the magnitude of the ACh-induced vasodilation observed at the conducted sites after the treatment with NOS blockers revealed that NO contributes to the propagation of the response by the spread of the signaling triggered at the local sites or by the activation of eNOS along the vessel length (Figures 2, 3A). To discern between these two possibilities, we stimulated a short vessel segment by applying a pressure-pulse ejection (300 ms) of SNAP, an NO donor, via micropipette to induce a vasodilator response comparable to the NO-dependent vasodilator component activated by ACh (i.e., the difference between control and L-NAME + L-NA, observed in each experiment, of the data shown in Figure 2). As anticipated, SNAP elicited a rapid vasodilation at the stimulation site, which showed a time course similar to that achieved in response to ACh (Figure 4). However, the vasodilator response induced by SNAP decreased very fast with distance, showing a mechanical length constant of 0.2 ± 0.04 mm (Figures 4A,B). As the magnitude of the dilation may have not reached the threshold for triggering a regenerative-like propagation of the response, we extended the pressure-pulse ejection of SNAP from 300 to 700 ms to elicit a larger vasodilation. Although the increase in the intensity of the stimulation resulted in a vasodilation like that induced by ACh at the application site (Figure 4A), the response decayed along the vessel length just as observed with the lower (300 ms) SNAP stimulation (Figure 4B), and the mechanical length constant observed in these arterioles was 0.36 ± 0.06 mm. In contrast, the longitudinal propagation of the NO-dependent vasodilator component of the ACh-induced vasodilation was much stronger than the SNAP-initiated conducted response, especially from the 500 µm to the 1000 µm of the conducted sites (Figure 4C), which suggests that the ACh-elicited NO-dependent conducted vasodilation represents the activation of eNOS along the vessel length.

### EDH-independent regenerative conducted vasodilation

**FIGURE 5:**
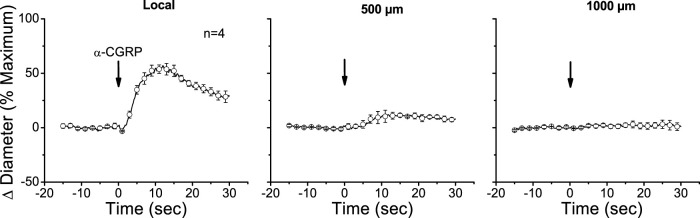
Time course of local and conducted vasodilation induced by the alpha isoform of calcitonin gene-related peptide (α-CGRP). A short segment of the cremasteric arterioles was stimulated with a pressure pulse-ejection of 1 µM α-CGRP via micropipette, and the resultant vasodilator responses were observed at the stimulation pipette site (local) and at locations 500, 1,000, and 2,000 µm upstream. Arrows indicate the time at which the stimulus was applied.

**FIGURE 6:**
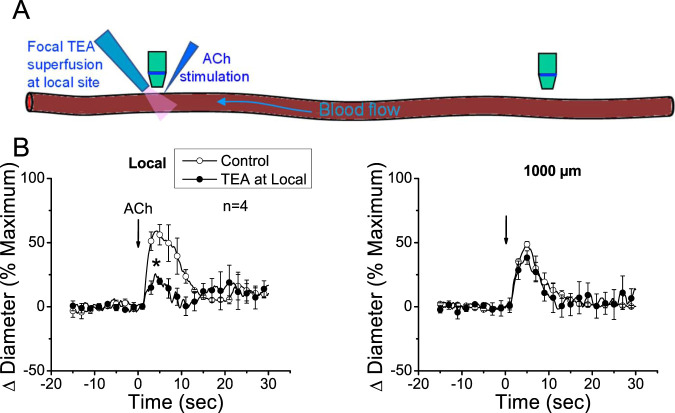
The conducted vasodilator response activated by ACh does not depend on the direct membrane hyperpolarization of endothelial cells generated at the stimulation site. **(A)** Schematic representation of the experimental procedure. **(B)** The time courses of the ACh-evoked vasodilation attained at the stimulation site (local) and 1,000 µm upstream were recorded under control conditions and during local application of 100 mM tetraethylammonium (TEA) via micropipette to prevent the Ca^2+^-activated K^+^ channel-mediated endothelial cell hyperpolarization. Note that the blockade of the EDH signaling-dependent vasodilation at the local site did not affect the conducted response observed 1,000 µm upstream. Arrows indicate the time at which the stimulus was applied. *P < 0.05 vs. control by one-way ANOVA plus Newman–Keuls *post hoc* test.

The apparent contribution of a locally activated NO-mediated vasodilator component to the conducted response initiated by ACh suggests that the regenerative-like conduction of the vasodilator response does not depend on the simple spread of the EDH signaling activated at the local site. To test this hypothesis, we evaluated the conduction of α-CGRP-induced vasodilation. α-CGRP is the main neurotransmitter of perivascular sensory nerve endings and is a potent vasodilator. Interestingly, although the vasodilation evoked by α-CGRP is associated with the hyperpolarization of the vessel wall (Nelson et al., 1990; Brain and Grant, 2004), the response activated at the stimulation site decreased rapidly along the vessel length (Figure 5). In addition, the contribution of EDH signaling to the conducted vasodilation was further tested by applying 100 mM TEA via micropipette to inhibit the activation of K_Ca_ channels exclusively at the stimulation site (Figure 6). In such conditions, TEA did not change the resting diameter of the vessel segment treated with this inhibitor (14.3 ± 0.3 vs. 16.6 ± 3.8). However, the ACh-induced vasodilation was strongly reduced in the TEA application site, and despite the clear effect on the direct response to ACh, this treatment did not affect the conducted vasodilation recorded 1,000 µm upstream from the local site (Figure 6), which is consistent with the propagation of a vasodilator signal, independent of K_Ca_ channels opening, which is coupled to the activation of the EDH signaling and NO production along the vessel length.

## Discussion

Control of the blood flow distribution depends on the coordination of the changes in vasomotor tone along the length of the arterioles and among the resistance vessels in the microcirculation (Segal, 2005; Segal, 2015; Figueroa and Duling, 2009). NO production plays a critical role in the tonic regulation of vasomotor tone, and conduction of vasomotor responses provides the bases for timing coupling of vascular function between different arteriolar segments and among arterioles with feed arteries (Gustafsson and Holstein-Rathlou, 1999; Segal, 2005; Figueroa and Duling, 2009). It is thought that conducted vasomotor responses rely on passive electrotonic spread of the changes in the membrane potential observed at the stimulation site through gap junctions connecting cells of the vessel wall, and in particular, gap junctions formed by connexin 40 have been demonstrated to provide a critical pathway for conduction of vasodilator signals through endothelial cells (Welsh and Segal, 1998; De Wit et al., 2000; Emerson and Segal, 2000; Figueroa et al., 2003; Figueroa and Duling, 2008; Jobs et al., 2012; Márquez et al., 2023). However, conduction of endothelium-dependent vasodilation does not seem to be consistent with the electrotonic mechanism since these responses have been observed to be conducted along the entire vessel without apparent decay, leading us to propose the participation of a regenerative, energy-dependent mechanism in the propagation of the endothelium-dependent vasodilator responses (Figueroa et al., 2007; Figueroa and Duling, 2008), similar to that described for electrical signals in neurons. In addition, consistent with this proposal, NO has been reported to inhibit the longitudinal conduction of vasodilator signals (Payne et al., 2004), suggesting that the regenerative mechanism involved in the propagation of the response is sensitive to NO. In agreement with these findings, our results indicate that the vasodilation activated by ACh is propagated for, at least, 2,000 µm, showing a much smaller decay in magnitude than that anticipated by an electrotonic conduction. Interestingly, inhibition of NO production with simultaneous application of L-NAME and L-NA converted the slightly decaying conducted response activated by ACh into a vasodilation that progressively increased along the vessel length, supporting the presence of an NO-sensitive mechanism of regenerative propagation of vasodilator responses.

NO production plays a critical role in the response to endothelium-dependent vasodilators, as well as in the regulation of vasomotor tone under resting conditions (de Wit et al., 1997; Figueroa et al., 2001; Moncada and Higgs, 2018). In line with this, although the treatment with L-NAME or L-NA alone evoked a modest reduction in the resting diameter, simultaneous application of both inhibitors resulted in a synergistic increase in vasomotor tone (i.e., vasoconstriction), highlighting the importance of NO in the regulation of microvascular function (Figure 1). In view that higher concentrations than 100 µM of L-NAME or L-NA do not result in a greater inhibition of NO production (Cohen et al., 1997; Boer et al., 2000) and may lead to a non-enzymatic NO formation from these NOS inhibitors (Moroz et al., 1998), we did not evaluate the increasing concentration of these blockers. As eNOS function depends on the subcellular location of the enzyme (Figueroa et al., 2002; Fulton et al., 2004; Church and Fulton, 2006), the synergistic effect observed with L-NAME and L-NA may be related to the uptake mechanisms and further intracellular distribution of the inhibitors. In endothelial cells, NO production is coordinated by dynamic subcellular targeting of eNOS between two functional pools of the enzyme: one associated with the trans-Golgi network and other located at caveolae, which are invaginated plasmalemmal rafts that function as signaling microdomains (Fulton et al., 2004; Jagnandan et al., 2005). Interestingly, the function of the trans-Golgi-associated eNOS pool depends on intracellular L-arginine, whereas the substrate supply of the caveolae-located pool of the enzyme is directly provided by the L-arginine influx (Mcdonald et al., 1997; Hardy and May, 2002; Zani and Bohlen, 2005). In this context, L-NAME can enter the cell through the plasma membrane, which may provide a preferential access to the trans-Golgi-associated eNOS pool. In contrast, L-NA uptake relies on the same amino acid transporter systems involved in L-arginine uptake, which, in addition to reducing the substrate supply to the caveolae-located eNOS pool may also favor the direct access of the inhibitor to the environment of the enzyme in this microdomain (Schmidt et al., 1993; Edwards et al., 1998; Boer et al., 2000). Thus, blockade of one eNOS pool may be compensated through an increase in NO production by the other eNOS pool, and therefore, we hypothesize that simultaneous inhibition of these two complementary eNOS pools potentiates the inhibition of NO production in a synergistic way, but the mechanisms involved in this process required further investigation.

The most relevant endothelium-derived vasodilator signals in resistance arteries are NO and EDH (Bakker and Sipkema, 1997; Bolz et al., 1999; Figueroa and Duling, 2009). Although the importance of NO production is widely recognized, the involvement of this signaling molecule in the generation of conducted vasodilation is debated (Hungerford et al., 2000; Segal et al., 1999; Budel et al., 2003; Welsh and Segal, 2017). Conduction of the changes in the diameter initiated by endothelium-dependent vasodilators, such ACh, is thought to rely on the electrotonic spread of the hyperpolarization of the vessel wall observed in response to EDH signaling at the stimulation site (Welsh and Segal, 1998; Gustafsson and Holstein-Rathlou, 1999; Emerson and Segal, 2000). However, the vasodilation evoked by ACh under control conditions was propagated over distances much longer than those predicted by the electrotonic model. In addition, the magnitude of the response increased along the vessel length after blocking NO production (Figures 2, 3), which is consistent with the finding that NO attenuates the conduction of vasoconstrictor responses along the length of mouse cremaster arterioles and the lack of decay observed in the propagation of the vasodilation activated by ACh from the local site (∼45%) to a distance of 2,000 µm (∼46%) in eNOS-knockout mice (Rodenwaldt et al., 2007). It must be noted that the increase in the conducted vasodilation was not related to the reduction in the local response attained in the presence of L-NAME and L-NA since the longitudinal decay in an ACh-elicited vasodilation of a similar magnitude showed characteristics exactly similar to those exhibited by the control response observed before the application of the NOS blockers (Figure 3). Therefore, these results suggest that, in addition to the EDH signaling-initiated conducted vasodilation, ACh also activates an NO-sensitive mechanism of regenerative propagation of vasodilator responses.

Although NO has been frequently thought to contribute only to the local response activated by endothelium-dependent vasodilators (Hungerford et al., 2000; de Wit et al., 2010; Welsh and Segal, 2017), Budel et al. (2003) found that the conducted vasodilation initiated by ACh is associated with the generation of an NO wave along the endothelium that was unmasked by a focal smooth muscle damage in the middle of the conduction pathway of the response. In line with this, our results indicate that blockade of NO production not only inhibited the vasodilation activated by ACh at the stimulation site, as expected, but also reduced the conducted response (Figures 2, 3). In this context, it is important to note that the vasodilation induced directly by NO (i.e., SNAP) decayed very rapidly with distance, in contrast to the NO-mediated vasodilator component that was propagated in response to ACh (Figure 4). Therefore, the reduction in the magnitude of the vasodilation observed at the conducted sites in the presence of L-NAME and L-NA suggests that the regenerative vasodilator signal initiated by ACh is coupled to NO production along the vessel length.

Interestingly, eNOS is a Ca^2+^-dependent enzyme, and then, the increase in NO production at a remote vessel segment from the stimulation site implies that the propagation of the ACh-induced vasodilator signal is associated with a mechanism that mediates an increase in [Ca^2+^]_i_, which, in addition to eNOS activation, may also trigger the myoendothelial signaling through the EDH pathway (Figueroa and Duling, 2009). It should be noted that endothelial cell hyperpolarization does not promote an increase in [Ca^2+^]_i_ in intact vessels, unlike what has been reported in the case of cell cultures (Ghisdal and Morel, 2001; Cohen and Jackson, 2005; McSherry et al., 2005; Stankevičius et al., 2006). Therefore, the increase in [Ca^2+^]_i_ attained at the conducted sites must be activated by a mechanism different from the simple membrane hyperpolarization. Consistent with this hypothesis, inhibition of EDH signaling activation, exclusively, at the ACh application site by micropipette-mediated focal superfusion of TEA did not affect the conducted vasodilation generated 1,000 µm upstream from the stimulation site (Figure 6). In these experiments, a TEA concentration of 100 mM was used in the superfusion micropipette; although this concentration may seem high, it must be noted that drugs applied via a micropipette are mixed with the bath solution, leading to, at least, a 10-fold reduction in its original concentration (Figueroa et al., 2007), which results in a concentration suitable for the inhibition of SK_Ca_ and IK_Ca_ channel activity. However, although TEA is a known K_Ca_ channel blocker, these results should be confirmed using specific inhibitors of SK_Ca_ and IK_Ca_, in combination with direct measurements of membrane potential.

The regenerative propagation of an electrical signal, different from K_Ca_-dependent hyperpolarization, coupled to an increase in [Ca^2+^]_i_ evokes the long-distance communication inherent to the information processing system observed in the nerves, and interestingly, the expression of voltage-dependent Na^+^ and Ca^2+^ channels has been detected in resistance arteries of mouse and other species (Gordienko and Tsukahara, 1994; Gosling et al., 1998; Walsh et al., 1998; Traub et al., 1999; Figueroa et al., 2007; Lillo et al., 2021). Therefore, we hypothesize that the stimulation of endothelial cells with ACh triggers a regenerative, conducted vasodilator signal that is mediated by the activation of voltage-dependent Na^+^ (Na_v_) channels and rapidly propagates along the endothelium through gap junctions. The Na_v_-mediated conducted electrical signal is transduced into vasodilation by the activation of the isoform Ca_v_3.2 of T-type, voltage-dependent Ca^2+^ channels and the subsequent initiation of the Ca^2+^-dependent activation of NO production and Ca^2+^-activated endothelial cell K_Ca_ channel-mediated smooth muscle hyperpolarization, as previously demonstrated in the case of the conducted vasodilation activated by focal electrical field stimulation (Figueroa et al., 2007). In addition to Ca_v_3.2 channels, the activation of the reverse mode of the Na^+^–Ca^2+^ exchanger may also contribute to the increase in [Ca^2+^]_i_ (Lillo et al., 2018). Consistent with this hypothesis and the results of the present work, it has been shown that NO-mediated S-nitrosylation inhibits the activity of Na_v_ channels (Bielefeldt et al., 1999; Renganathan et al., 2002). Although the participation of this mechanism in the response initiated by ACh remains to be determined, it must be noted that Na_v_ channels may be activated by small depolarizing currents, such as those generated through TRP channels in response to endothelium-dependent vasodilators (Vincent and Duncton, 2011).

In summary, the results of the present study are consistent with the hypothesis denoting that ACh, in addition to the local response, triggers the regenerative propagation of a vasodilator signal coupled to a mechanism that leads to NO production and activation of the EDH signaling along the length of the arterioles. Interestingly, the ACh-elicited regenerative propagation mechanism of vasodilator responses is sensitive to NO, which appears to function as a negative feedback signaling of the conducted response, and consequently, the magnitude of the vasodilator responses observed along the vessel length increases after blocking the eNOS activity. Coordination of the changes in diameter among different segments of resistance arteries plays a central role in controlling the blood flow distribution and arterial blood pressure, and then, these findings may contribute to the understanding of the mechanism involved in the vascular dysfunction typically associated with the progress of cardiovascular-related diseases, such as hypertension and diabetes. However, the proposal that endothelium-dependent vasodilators activate the regenerative propagation of vasodilator signals through an NO-sensitive mechanism must be confirmed not only in mice but also in other species, through *in vivo* analysis of the electrophysiological characteristics associated with the conduction of vasodilator signals throughout endothelial cells.

## Data Availability

The raw data supporting the conclusions of this article will be made available by the authors, without undue reservation.
